# A rare case of primary cutaneous follicle centre lymphoma presenting as a giant tumour of the scalp and combined with JAK2V617F positive essential thrombocythaemia

**DOI:** 10.1186/2050-7771-2-7

**Published:** 2014-04-01

**Authors:** Yordanka Tirefort, Xuan-Cuong Pham, Yasmine Lucile Ibrahim, Thomas Pierre Lecompte, Thomas Matthes, Christa Prins, Begonia Cortes, Michael Bernimoulin, Yves Chalandon, Kaveh Samii

**Affiliations:** 1 Department of Haematology, University Hospital of Geneva, Rue Gabrielle-Perret-Gentil 4, 1211 Geneva, Switzerland; 2 Department of Dermatology, University Hospital of Geneva, Rue Gabrielle-Perret-Gentil 4, 1211 Geneva, Switzerland; 3 Department of Clinical Pathology, University Hospital of Geneva, Rue Gabrielle-Perret-Gentil 4, 1211 Geneva, Switzerland

**Keywords:** Primary cutaneous follicle centre lymphoma, PCFCL, Essential thrombocythaemia, JAK2V617F, Myeloproliferative neoplasm

## Abstract

Primary cutaneous follicle centre lymphoma (PCFCL) is a rare cutaneous B cell lymphoma in middle-age adults with excellent prognosis. Here we present a case of a patient with a PCFCL in the form of a giant tumour of the scalp in combination with a myeloproliferative neoplasm, JAK2V617F positive essential thrombocythaemia. This case may be of interest because of the favourable outcome in spite of the large size of the PCFCL, the rare combination with essential thrombocythaemia and because it contributes to discussion on the role of JAK2 mutation in such patients.

## Background

Lymphoproliferative neoplasms (LPN) are a heterogeneous group of tumours of the lymphoreticular system that might manifest themselves as extranodal lymphomas, localized in organs or tissues. Primary cutaneous B-cell lymphoma is a distinct type of extranodal lymphomas with a particular clinicopathologic presentation [1]. Primary cutaneous follicle centre lymphoma (PCFCL) is the most common variant of cutaneous B-cell lymphomas, representing about 18% of all primary cutaneous lymphomas [2]. PCFCL usually manifests itself as variably sized, solitary or grouped erythematous lesions, localized in the head, neck, trunk, and upper extremities or, as in our patient, as multifocal lesions. Treatment almost always leads to complete remission but cutaneous relapses may occur.

Essential thrombocythaemia (ET) is a clonal myeloproliferative neoplasm (MPN) and JAK2V617F mutations are found in approximately 50% of ET patients. Only a few cases of concomitantly occurring MPN and LPN have been reported [3-8]. Laurenti et al. published a multicenter retrospective study on the association of chronic lymphocytic leukemia (CLL) and concomitant MPN [9] but at present, no systematic analysis for MPN-LPN disease association is available and underlying pathogenic mechanisms remain unclear. Here we present a patient with ET and a giant PCFCL tumour of the scalp treated with chemo- and radiotherapy.

## Case presentation

**Figure 1:**
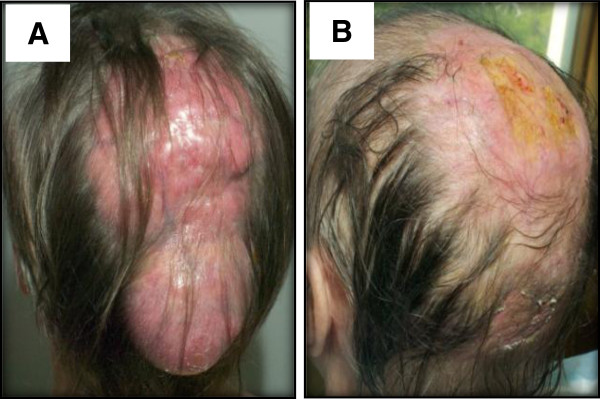
Multinodular mass on the scalp (A) and spectacular response after the second chemotherapy course (B).

**Figure 2:**
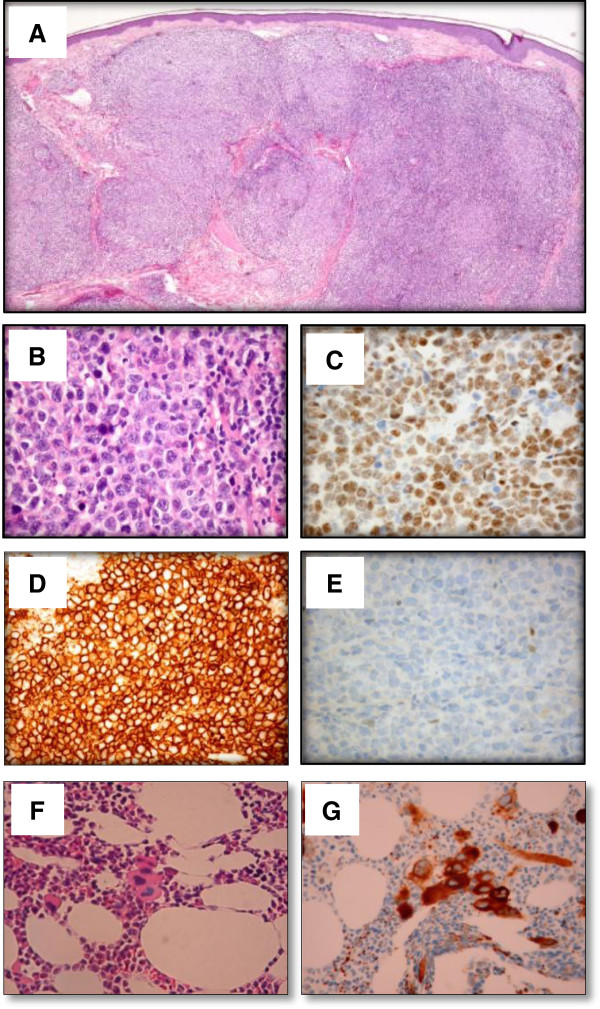
**Histopatological fingings.** Biopsy of the mass of the scalp showing a dense and diffuse dermal infiltrate with nodular growth pattern **(A)** consisted of large atypical lymphocytes (H&E) **(B)**, which are positive for CD20 **(C)** and Bcl6 **(D)** and negative for Bcl2 **(E)** on immunohistochemical staining. Bone marrow biopsy showing a hypercellular bone marrow with proliferation of megakaryocytes with hyperlobulated nuclei, sometimes in loose clusters (H&E) **(F)**, positive for CD61 on immunohistochemical staining **(G)**.

**Figure 3:**
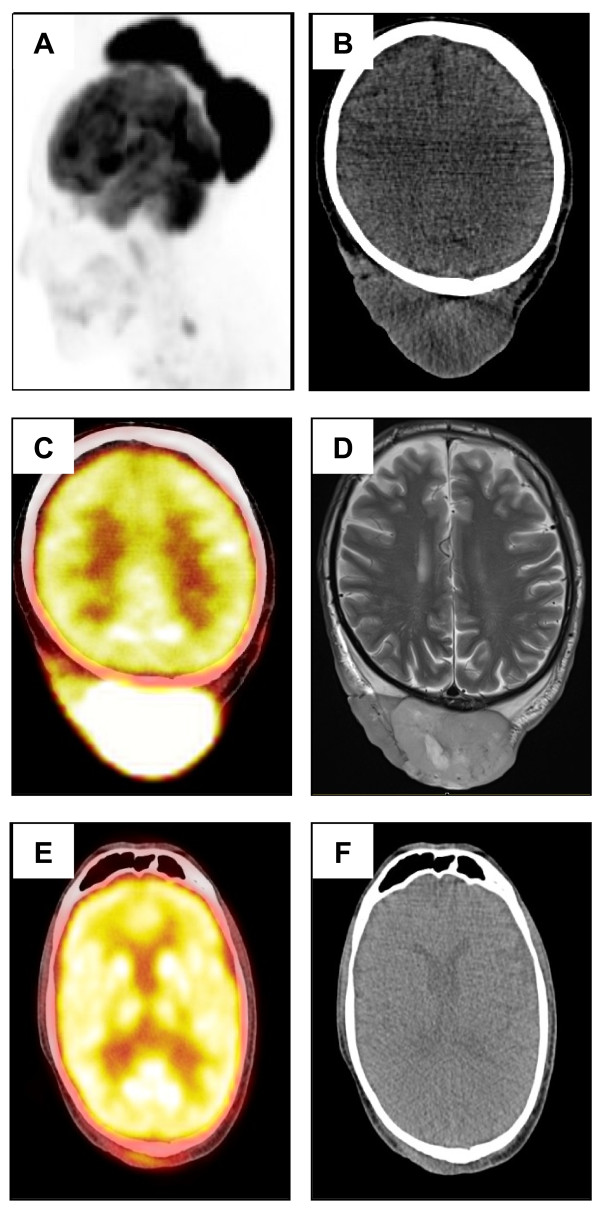
**Radiological investigations showing the lesions before treatment and their evolution after the treatment.** MRI at diagnosis **(A)**, **(B)**; PET-CT at diagnosis **(C)**, **(D)**; PET-CT and MRI after 2 cycles of R-CHOP **(E)**, **(F)**.

A 56-year-old man with an unremarkable medical history developed a multinodular mass on the scalp reaching 19 × 16 × 4 cm over 18 months (Figure 1). Similar lesions appeared on the face. Skin biopsies showed a dense and diffuse infiltrate throughout the dermis and the subcutis of CD20, CD79a and Bcl-6 positive, CD10 and IRF4 negative, large lymphoid cells with a focal nodular growth pattern (Figure 2), an image typical of cutaneous follicle centre lymphoma with a diffuse growth pattern. IgH/Bcl-2 rearrangements were not detected using FISH. Cross-sectional PET/CT and MRI imaging did not reveal any evidence of systemic spread of the lymphoma except for a suspicious spinal lymph node on the right side of the neck (Figure 3). According to ISCL/EORTC classification of cutaneous lymphomas [1], a diagnosis of PCFCL, stage T2bN1M0 was made. Platelet count was 772 × 10^9^/L, other blood counts were normal. LDH was normal and there was no splenomegaly. Bone marrow biopsy revealed a moderate hypercellularity with a marked proliferation of mature megakaryocytes with hyperlobulated nuclei, sometimes in loose clusters, without increase in reticulin fibres (Figure 2). Quantitative real-time PCR showed that 24% of the nucleated cells in the blood carried the JAK2V617F mutation, while scalp biopsies were only weakly positive (<3%), probably due to granulocyte contamination. Screening for HIV, EBV, HBV and HCV was negative.

### Treatment

Because of the exceptional size of the tumour, its location and possible nodal involvement, the patient received systemic chemotherapy consisting of 4 cycles of Rituximab combined with Cyclophosphamide, Adriamycin, Vincristine and Prednisone every 21 days (R-CHOP-21) followed by consolidation radiotherapy. After the second R-CHOP cycle, we observed a spectacular response with 90% reduction of tumour mass (Figures 1B, 3E, 3F). At the time of writing, 2 years after diagnosis, the patient is still in remission. The chemotherapy did not induce a remission of ET and platelet counts remained stable at 600 – 700 × 10^9^/L at 17 months after the last cycle of chemotherapy. Because the patient was without clinical symptoms of ET, no treatment other than a low dose prophylactic aspirin was given.

## Discussion

We present herein a patient with PCFCL and a concomitant ET, two neoplasms of which the relation is still under debate [3-8]. For instance, Palandri *et al*. reported that 8 of 499 ET patients (1.6%) also suffered from LPN [6], while Rumi at al. found a 2.79-fold higher risk of developing LPN in patients with MPN than in the general population [8]. Similarly, Vannucchi *et al*. found a significantly increased risk of developing LPN in patients with MPN harbouring the JAK2V617F mutation, that varied from 2.7 fold for any LPN and ET, 4.2 fold for any LPN and PV to 12 fold in case of CLL and both ET and PV [7]. Because the JAK2V617F mutation was found in 2 of 3 LPN examined, they suggested that MPN and LPN might originate from a common lymphoid-myeloid progenitor. However, other studies did not report JAK2 mutations in the majority of LPN which underlines that JAK2V617F might only be one of the perhaps several factors favouring the genetic instability that predisposes to lymphoid and to myeloproliferative neoplasms as well [3,10,11].

We have treated our patient with R-CHOP-21, which led to complete remission of the PCFCL without impacting the ET. This is reminiscent of the findings of Laurenti *et al*. who found that the course of MPN was not influenced by treating the coexisting CLL and that treating the MPN with hydroxyurea or imatinib did not impact the course of the LPN [9]. Although Palandri *et al*. did not find an increase in thrombotic events in their cohort of patients with concomitant ET and LPN [6], conventional chemotherapy for lymphoma might increase this risk considerably [12]. In the future, alternative treatment modalities, as those targeting the JAK/STAT pathway, might be useful because of their potential interest for treatment of both disorders in MPN-LPN patients. Downstream targets of the JAK2 pathway such as the signal transducer and activator of transcription (STAT) proteins, the Ras/Raf/mitogen-activated protein kinases (MAPK), and the phosphatidylinositol-3 kinase (PI3-K)/Akt pathways are often overexpressed in lymphoma subtypes such as primary mediastinal B-cell- and Hodgkin lymphoma [13-15]. Whether this is also true for the patient’s PCFCL is not known. Unfortunately, we have not been able to test whether the JAK2V617F mutation in our patient had led to a sustained activation of the JAK2 pathway. If so, it would make sense to treat future MPN-LPN patients with the drugs targeting oncogenic JAK/STAT pathways that are currently under investigation for the treatment of myeloid and lymphoid neoplasms [16,17]. A recent phase I trial has already demonstrated a potential efficacy of the JAK2/FLT3 inhibitor Pacritinib (SB1518) for the treatment of different types of lymphoma reducing the tumour mass in half of the 31 patients enrolled with minimal haematological toxicity [18]. Furthermore, phase II studies of SB1518 in patients with myelofibrosis [19,20] are now ongoing and if successful, this would certainly provide a rationale for its use in patients with concurring myeloid and lymphoid malignancies.

## Conclusion

The present case is of interest because of the unusual presentation of PCFCL as a giant tumour of the scalp with multifocal lesions but nevertheless excellent evolution. To our knowledge, this is the first report of a patient with MPN, JAK2V617F positive ET, and PCFCL. The standard R-CHOP-21 therapy was successful for the PCFCL but this treatment did not influence the evolution of ET. In the future, therapies targeting JAK/STAT pathways that have shown potential for treatment of MPN as well as for lymphoma, may be considered an alternative.

## Consent

Written informed consent was obtained from the patient for publication of this case report and the accompanying images. A copy of the written consent is available for review by the Editors-in-Chief of this journal.

## Competing interest

The authors declare no competing financial interest.

This case has been presented as a poster at the 81th Annual assembly of Swiss Society of Internal Medicine in May 2013 in Basel, Switzerland.

## Authors’ contributions

YT, XCP, YLI, TM and CP diagnosed the patient. YT, MB, KS and BC treated and are following the patient. YT, TPL and YC wrote the manuscript. KS supervised the work. All authors reviewed the manuscript. All authors read and approved the final manuscript.
